# Toxicology and Legal Medicine

**Published:** 1859-09

**Authors:** 


### TOXICOLOGY AND LEGAL MEDICINE.

1. Application of Crystals of the Blood to Legal Medicine. By *Prof. Bryk*, of Krakau.—By heating dried blood with organic acids, and principally with concentrated acetic acid, it is easy to obtain hematin crystals, even when the quantity of blood is excessively small. By this crystallization, blood-stains can be recognized, which could not be distinguished by the microscope, from the characters of the globules. It is sufficient to reduce a small part of the matter, which composes the suspected spot, to a fine powder, to put it on a glass plate, to cover it with a small glass, then to add some drops of crystallizable acetic acid, and to dry it slowly on the sand-bath. After having washed the preparation, it is put under the microscope. We then discover crystals in the form of prisms or rhomboid plates, the larger angles of which are commonly round, and which have a brownish-red, brown, or yellow color. These crystals are less abundant when the blood is putrefied or mixed with pus. When there is an admixture of pus, they are more easily obtained by previously treating the stains with ether.

When these crystals are treated with a concentrated solution of caustic potassa, they change gradually from the green to a purplish-red color; with concentrated sulphuric acid, they assume a greenish-yellow, then a reddish-brown, impure violet, brick-red, and finally a rosy color; this character suffices to distinguish them from all other stains. Those which are produced by bile, react, however, in the same manner with sulphuric acid, but they are not modified by potassa.

These changes of color are very easily obtained, by applying the same reagents directly on the stuffs (not colored) which show the blood-stains, even when they have been washed, provided they retain some atoms of hematin.

The knowledge of these facts is owing particularly to MM. Schmidt, Rose, and Brücke. Professor Bryk adds, that blood-stains readily lose these characters, and furnish no more crystals after having remained for some time on wood.

The character of crystallization, first pointed out by Feishmann, has already found, in the hands of Brücke and Virchow, medico-legal application. This means, however, permits us to recognize only the presence of blood; it furnishes no information in reference to the species of animals from which the blood comes.—(*Wiener Medizinisch. Wochenschrift*, Nos. 42, 44, and 45, 1858.)

2. SOUTH AMERICAN ARROW-POISON. By *Wm. R. Hammond, M.D., U. S. A.*, and *S. W. Mitchell, M.D.*—The following is a brief *résumé* of a long paper which appeared in the American Journal of the Medical Sciences for July, 1859, upon carroval and vao, two new varieties of woorara, or curare, as it is termed in France. The authors preface their own observations with an elaborate history of woorara, and of the chemical and physiological labors already bestowed upon it by Rolliker, Bernard, Pelikan, Velpeau, Brodie, etc. They then proceed to describe the physical characters, chemistry, physiological relations, and toxic peculiarities of the two new, or hitherto unknown varieties of woorara which have fallen into their hands. They thus examine separately the two substances carroval and vao, and state in detail the reasons which induce them to consider vao as but a weaker carroval: or else, if we comprehend them aright, as bearing to carroval some such relation as brucia bears to strychnia, or cinchonia to quinia.

Both poisons yielded, to appearance, one and the same essential principle, a new alkaloid, which the authors have termed carrovalia. This substance, whether from vao or carroval, was of a deadly potency; since, however, the authors explain that their supply of the poison was not sufficient to enable them to complete the chemistry of the subject, we shall await some further developments in regard to the tests for the new alkaloid. Although the two poisons were studied separately, it is unnecessary here to view them apart, since it has been shown that the chief dissimilarity is one of degree, and not of kind.

The new woorara, then, according to Drs. Hammond and Mitchell, differs remarkably from that long known to the toxicologists of Europe. Thus the woorara of our authors kills by paralyzing the heart; while the other, or European woorara, destroys by paralyzing the nerves of motion throughout the body. This singular difference completely isolates the carroval and vao poisons from all other woorara hitherto examined, and approximates them in character to the upas poison, so ably investigated by Professor Rolliker, whose toxicological labors are as yet little known or appreciated in this country.

We refer to the following "conclusions," as setting forth what the authors conceive themselves to have proved in regard to these poisons, premising that as they seem to have been finally of opinion that carroval and vao are nearly allied, if not identical, we have thought it unnecessary to copy both sets of conclusions. We have appended to each conclusion, in brief form, a statement of the proofs in favor of the proposition set forth.

1. Carroval and vao are capable of being absorbed from the areolar tissues, and from the stomach of warm and cold-blooded animals. When given by the stomach, both poisons are more apt to cause convulsions than under other circumstances. The larger part of the absorption experiments were made with vao only. From these it was ascertained, say our authors, that it (vao) is also absorbed by the stomach, œsophageal canal, rectum, and skin of cold-blooded animals, (frogs,) with a degree of rapidity which varies; and is rapid or slow as the animal is ill or well supplied with water.

Warm-blooded animals absorb vao from the stomach and intestines when they are fasting, but suffer no ill effects when the vao is given during digestion. That this protection is not due to a mere mixture of the vao with the food of the full stomach, is shown by the fact that rabbits, whose stomachs are always more or less distended with food, are protected only when, owing to the entry of fresh food, digestion becomes active.

Both carroval and vao arrested the heart's action. This was shown by exposing the hearts of frogs, and then inserting the poisons under the skin, when the heart began to beat more strongly, then became paralyzed in parts, and finally stopped, and was found to be so dead, that in most cases no galvanic or other stimulus could induce a single pulsation. After this, the frogs thus poisoned leapt about until the cessation of the circulation produced its usual effect, in paralyzing first the sensitive and then the motor nerves. These secondary effects our authors have shown to follow any arrest of circulation in the frog. Thus, when the heart was tied, sensation and motion disappeared in an hour, even where no poison was given. Both carroval and vao were found to arrest very early the motion of the lymph hearts, and both of them undoubtedly lessened the duration of muscular irritability; while the ordinary woorara either does not affect it, or, according to Bernard, lengthens its duration.

When these poisons are administered to warm-blooded animals, death usually occurs without convulsions, except in the cat, where they nearly always are seen. In animals of this class, the arrested circulation stops the aeration of the blood, so that the checked respiration is to be looked upon as a consequence and not a cause of the injury to the cardiac functions. This was also illustrated in the case of the alligator, whose respiration continued long after the heart had ceased to move. It was also found that artificial respiration was of no value as a means of relief, as might have been suspected from the fact that the poisons destroyed the muscular irritability of the heart.

The authors were unable to discover that these poisons altered the blood, or affected the ciliary movements. They are of the opinion that the varieties of arrow-poison examined by them are of a purely vegetable origin, and contain no trace of the venom of serpents.

3. ON A NEW POISON FROM THE INTERIOR OF CHINA. By *Robert Christison, M.D.*, Professor of Materia Medica in the University of Edinburgh.—About eighteen months ago I received from China a copy of a newspaper printed at Shanghai, giving an account of a formidable poison, said to be used far west, in the interior of the country, for killing game and in warfare.

According to this account, springs had been invented for using it against the British invaders; and as these had been found to answer against goats, much was expected from its aid at Canton. But, unluckily, the sudden peace nipped the scheme in the bud. That the poison might have been employed with deadly effect is very probable, if only one-half of what was said of it be true; for the account in the *Northern China Journal* (April 4, 1857,) represents it to be so virulent that "instant death is inevitable from the slightest abrasion" by an arrow tipped with it. The operator, who prepares it in the shape of an extract, goes on concentrating and strengthening it until a small animal, whose skin is punctured with it, dies instantaneously: not until then, it seems, does he cease to boil down his extract. In the mountainous regions of Chihkiang large game is killed by means of arrows whose neck is encircled by the poison. Even tigers are killed in this way by the Funghwa hunters. Should a limb be struck, the beast writhes awhile before expiring; but should the arrow "hit the body, he leaps forward, staggers, and immediately falls down dead."

This is marvelous enough. But it is also said to be of a singularly volatile or progressing nature. For if the poison "be applied to blood trickling from a little wound, even though only to the lower end of the stream, the blood is rapidly blackened along its whole length; and if the stream be continuous with the wound, the subtle poison will enter, and occasion death."

This poison is the extract of a root called *Tsau-wú;* and the plant is represented to be a perennial creeper inhabiting the central provinces of the Chinese Empire. It is added, that the extract is sometimes applied, as a practical joke, to the tongue of the unwary, "in whom it excites a keen sense of formication."

In the beginning of January last I received specimens relative to this extraordinary poison from Dr. D. J. Macgowan, an American physician residing at Ningpo. The specimens included not only the poison itself, but likewise some leaves and roots of the plant which yields it. The materials are scanty; yet they are sufficient to enable me to ascertain the source of the poison pretty nearly.

The leaves present characters common to several plants of the Ranunculaceous family. Only one of the roots, which are but four in number, is entire; but even the structure of this alone, taken along with its singular impression on the organs of taste, is sufficient to prove that the plant is a species of *Aconitum*, or Monkshood. All the species of Aconitum that I have examined spread by root in the following manner, of which the *A. Napellus* may be taken for an example. During winter there is nothing to be seen but an under-ground tuber, tap-shaped in all the poisonous species, with rootlets from the lower extremity, and an incipient leafy bud at the crown. Early in spring the bud begins to shoot out into the future stem, which attains its full stature toward the close of summer, or a little later. During its progress, early in summer, a little knot forms near where the crown and stem meet; and from this is gradually produced a new tuber exactly like the primary one, parallel to it and connected with it by a small band. Near the close of autumn, when the seeds of the plant are mostly ripe, both tubers seem equally vigorous, and of equal size; but the new one is firm, and continues plump on being dried, while the old one is more watery, shrivels much in drying, and, in fact, soon rots and disappears in the ground, leaving the new tuber to perpetuate the plant next season. Now, one of the Chinese roots shows a portion of the stem, with its tap-shaped, somewhat shriveled tuber at the bottom; and, attached to the crown of this tuber, a plump, firm, tough, tap-shaped tuber of the same size. No one familar with the Aconites can fail to recognize in this Chinese specimen a miniature representation of the root of the *A. Napellus* of Europe, and *A. Ferox* of the Himalayas.

The likeness is maintained in the very singular impression produced by this root upon the organs of taste. A little bit, no bigger than a pin's head, carefully chewed, while it is held in one spot between the tip of the tongue and the lips, produces intensely that strange combination of numbness and tingling which characterizes so remarkably all the poisonous species of the known Aconites. It is an impression so peculiar, that I do not know any plant, not an Aconite, which produces a sensation like it, except the *Delphinium Staphysagria*, or Stavesacre,—another plant of the *Ranunculaceæ*, which, with its whole genus, presents close natural resemblances to the Monkshoods.

I have no doubt, therefore, that the Tsau-wú, or Wú-tsau,—for the name is variously given by my informant in his letter and in the labels,—is the root of a Monkshood. It is probably a new species; for, though like a diminutive *A. Napellus*, it is much whiter and more amylaceous in its interior, and yet evidently, from its taste, much more active. It is by much the smallest aconite root of the poisonous species; for the whole tuber is scarcely an inch long, and delicate in its structure.

It only remains to be seen whether the poison is really prepared from this root. The extract sent to me is still soft, and evidently made with great care and skill. The most minute portion causes in the tongue and lips precisely the same impression as the Wú-tsau root, but with extraordinary intensity. The Wú-tsau is in it therefore; and nothing more is wanted to make a most virulent and efficacious arrow-poison.

I must here enter my protest, however, against some of the statements which have been made to Dr. Macgowan by his Chinese informants, as to the action of this extraordinary poison—and, indeed, against the facility of belief among travelers generally respecting the effects of arrow-poisons used by barbarous and semi-barbarous tribes of men. The Wú-tsau cannot by any possibility travel up a stream of blood, against the current, and into a blood-vessel. It cannot cause instant death in any animal. No arrow-poison can cause instant death. All poisons which act through a wound must take a little time to act; because they act through absorption into the blood upon distant parts—the brain, the spine, or the heart; and a solid poison cannot thus reach its destination in sufficient quantity all in a moment, even though the arrow pierce a blood-vessel.

It is possible to account for the frequent error of travelers as to the alleged instantaneousness of the action of arrow-poisons. The lapse of time is, in the first instance, apt to be overlooked or understated. But, further, if such formidable poisoned arrows as those used by the negroes of the Upper Gambia, or the Macusi tribe at the sources of the Essequebo, be struck into the trunk of even a large animal by means of their strong bows, the arrow may quite well reach a vital part, and thus arrest motion at least, if not occasion very speedy death, merely as an arrow, by mechanical violence. Small animals and birds may be thus brought down suddenly even by the little wooden darts shot through blowing-tubes by the Macusi natives of the Essequebo, as well as other wild tribes. The Macusis use the deadly Urari* poison. But the dart may kill as an arrow quite as well as through means of the poison, should it hit the trunk of a small animal; for its force is surprisingly great. With very little practice, I can blow the little, light, wooden dart 180 feet; strike it at 60 feet so firmly into a board that it cannot be removed unbroken; and transfix with it, at a short distance, a fir board a fourth of an inch in thickness, or thirty folds of cartridge-paper.

I will also venture to take exception to the frequent propensity of travelers and others to magnify savage skill in the manufacture of such poisons, at the expense of civilized ingenuity. The Urari of the Essequebo, the Upo or Upas of Java and Borneo, the Wú-tsau of China, are potent poisons, no doubt. But in potency they never will stand comparison with several of the pure principles which the chemistry of civilized nations has detached from poisonous vegetables. Any tribe of men, compelled by circumstances to obtain their food by shooting game with poisoned arrows, would profit greatly could they substitute digitaline, aconitina, conia, strychnia, and other pure principles of plants, for their own cruder extractiform poisons. These principles, indeed, might be so used as to deal destruction to the very largest animals on the face of the globe.—(*Edinburgh Medical Journal*, April, 1859.)

---

* Generally corrupted by English writers into *Wourali*, and by the French into *Curare*. Schomburgk, the best authority, says that the Macusis who make it call it invariably *Urari*.

